# CRF Mediates Stress-Induced Pathophysiological High-Frequency Oscillations in Traumatic Brain Injury

**DOI:** 10.1523/ENEURO.0334-18.2019

**Published:** 2019-05-10

**Authors:** Chakravarthi Narla, Paul S. Jung, Francisco Bautista Cruz, Michelle Everest, Julio Martinez-Trujillo, Michael O. Poulter

**Affiliations:** 1 Robarts Research Institute, Schulich School of Medicine, University of Western Ontario, London, Ontario N6A 5K8, Canada

**Keywords:** traumatic brain injury, stress, voltage sensitive dye imaging ripples, epilepsy, rat

## Abstract

It is not known why there is increased risk to have seizures with increased anxiety and stress after traumatic brain injury (TBI). Stressors cause the release of corticotropin-releasing factor (CRF) both from the hypothalamic pituitary adrenal (HPA) axis and from CNS neurons located in the central amygdala and GABAergic interneurons. We have previously shown that CRF signaling is plastic, becoming excitatory instead of inhibitory after the kindling model of epilepsy. Here, using Sprague Dawley rats we have found that CRF signaling increased excitability after TBI. Following TBI, CRF type 1 receptor (CRFR_1_)-mediated activity caused abnormally large electrical responses in the amygdala, including fast ripples, which are considered to be epileptogenic. After TBI, we also found the ripple (120–250 Hz) and fast ripple activity (>250 Hz) was cross-frequency coupled with θ (3–8 Hz) oscillations. CRFR_1_ antagonists reduced the incidence of phase coupling between ripples and fast ripples. Our observations indicate that pathophysiological signaling of the CRFR_1_ increases the incidence of epileptiform activity after TBI. The use for CRFR_1_ antagonist may be useful to reduce the severity and frequency of TBI associated epileptic seizures.

## Significance Statement

The combination of traumatic brain injury (TBI) and stress is known to increase the likelihood of posttraumatic epilepsy (PTE). Here, we show after moderate TBI that corticotropin-releasing factor (CRF) increases the excitability of a number of limbic regions in the rat. In particular a tail pinch stress causes epileptiform activity that is characterized by the occurrence of fast ripples that are phase amplitude coupled to θ rhythm. This activity is blocked by CRF receptor 1 (CRFR_1_) antagonist. Our data provide a possible mechanism by which stress exacerbates epileptiform activity.

## Introduction

Traumatic brain injury (TBI) refers to injuries that include concussions, impacts to the head, blast injuries as well as others (Maas et al., 2008). It affects a wide spectrum of individuals in the population regardless of age, background, and health status and is, therefore, a critical public health problem throughout the world (Langlois and Sattin, 2005; Tagliaferri et al., 2006; Maas et al., 2008). TBI is associated with the development of neurocognitive deficits, such as impaired attention and depression (30–70% of people with TBI develop depression; Roozenbeek et al., 2013). In particular, mild TBI has been shown to predispose people to psychiatric disorders, such as anxiety, depression, mood disorders, and disruptive behaviors (Curvis et al., 2016; Emery et al., 2016). Among these individuals, TBI is also a leading cause of acquired epilepsy (Campbell et al., 2014; Wilson et al., 2017; Xu et al., 2017; Zimmermann et al., 2017). Compared to general population, individuals sustaining TBI have on average a 3-fold higher risk of developing epilepsy (Martin et al., 2014; Rehman et al., 2015; Chen et al., 2017). Epilepsy has a substantial impact on the quality of life of affected individuals, and in the United States alone, it has an estimated cost of approximately $13 billion each year (Begley and Durgin, 2015). Importantly, heightened anxiety is a precipitating factor for seizure genesis after TBI (Juengst et al., 2017). How this occurs is not understood, but it is clear that a cascade of molecular, electrophysiological, and cellular changes initiates myriad outcomes that result in the development of posttraumatic epilepsy (PTE; Pitkänen et al., 2014; Lucke-Wold et al., 2015). Thus, a better understanding of how stressors and TBI interact to increase the onset of PTE is required. In particular, there are numerous case studies linking TBI to posttraumatic stress disorder (PTSD; Levin et al., 2001; Greenspan et al., 2006; Gaylord et al., 2008; Hoge et al., 2008). The risk of development of epilepsy later in life is higher in patients with PTSD compared to patients without PTSD (Chen et al., 2017).

Corticotropin-releasing factor (CRF), a 40-amino acid neuropeptide, is well known to be implicated in stress and anxiety disorders. It is released in various brain regions during stress and mediates endocrine/behavioral responses. In normal rats, CRF dampens the excitability of piriform cortex (PCtx; Narla et al., 2015). This effect is mediated by CRF receptor 1 (CRFR_1_) signaling through the activation of the G-protein, Gα_q/11_. CRF exacerbates seizures in a kindling model of epilepsy through the activation of CRFR_1_ in PCtx (Narla et al., 2016). After kindling, CRFR_1_ signals the activation of a different G-protein, Gα_s_, and in this case, CRF causes the excitation of overall PCtx circuitry. Thus, epilepsy altered “the polarity” of a stressor signal increasing the excitability of the PCtx. Here, we hypothesize that after TBI, CRF activity changes in a manner that supports the generation of epileptiform activity during stress. Indeed, we show that TBI alters widespread CRF activity, enhancing network excitability (as in kindling). CRFR_1_ activation was responsible for the generation of pathophysiological high-frequency oscillations (HFOs; >250 Hz; fast ripples) and these oscillations were phase-locked with a very large increase in the power of θ activity. Also, we show that CRF activation has a similar response in the kindling model enhancing the incidence of fast ripples.

## Materials and Methods

Procedures were performed in accordance with the guidelines of Canadian Council of Animal Care and approved by the University of Western Ontario Animal Care committee.

### TBI procedure

Adult male Sprague Dawley rats weighing 150–180 g were used in all of our experiments. They were housed individually with free access to food and water under a continuous 12/12 h light/dark cycle. Animals were anesthetized with ketamine-medetomidine hydrochloride combination and placed in a stereotaxic frame for positioning under a pneumatic impactor (Precision Science Instruments). The animals were placed under 2% isoflurane to maintain the anesthesia throughout the surgery. Scalps were shaved, and rats were placed in a stereotactic apparatus. Eye ointment (LacriLube) was applied throughout the procedure to prevent irritation and drying. The scalp was scrubbed with povidone-iodine, a bacteriostat, and 70% ethanol, respectively, with sterile cotton swabs. A 6- to 8-mm longitudinal incision was made in the scalp, and sterile tissue clamps were used to hold the incision open to expose the skull. The fascia was scored and gently removed with a scalpel. Stereotactic coordinates were determined, and a craniotomy was performed at 2.0 mm anterior to bregma, 4.8 mm lateral to the midline. A 1-mm hole was drilled through which the piston was introduced. Animals received a unilateral injury to the surface of the brain at above-mentioned coordinates. The injury parameters consisted of 2.5-mm cortical compression at a speed of 3.5 m/s for 500 ms. Sham animals received a craniotomy but did not receive an impact to the brain. Following the impact, the skull cap was placed back in place using Vetbond tissue glue. Stereotactic coordinates were determined and electrodes were implanted at 2.6 mm posterior to bregma, 4.8 mm lateral to the midline, and 8.0 mm ventral (Paxinos and Watson, 1986). The electrodes were made of two twisted strands of 0.127-mm diameter diamel-insulated nichrome wire and were attached to male amphenol pins. The electrodes were implanted and secured to the skull with jeweler’s screws. The electrode assembly was fixed to the skull by dental acrylic cement (McIntyre and Molino, 1972). Animals were then removed from isoflurane anesthesia and placed in a clean cage. The temperature was maintained at 37°C with the use of a heating pad. Rats were not used for either slice experiments or *in vivo* recordings for a least one week after surgery. During this time rats were behaviorally monitored to ensure that they were eating and drinking normally and not exhibiting any sickness behaviors. All rats used for this study survived the surgery and recovered.

### *In vitro* experiments

#### Slice preparation

Animals were anesthetized with a ketamine-medetomidine hydrochloride combination and then perfused through the heart with an ice-cold artificial CSF (ACSF) in which sodium ions were replaced by choline ions. The brain was rapidly removed (<2 min) and then transferred into cold choline ACSF. Slices were prepared on vibratome; once sliced, they were warmed to 32°C for 30 min and then transferred to room temperature bath until they were needed for recordings.

#### Voltage-sensitive dye imaging (VSDI)

Brain slices from Sprague Dawley rats (six sham and six TBI) were used for the VSDI recordings. These were 800 μm thick and perfused on both sides during recordings. Slice were incubated in the voltage-sensitive dye Di-4-ANEPPS (D-199, Invitrogen Inc.) for 35 min. The stock solution of the dye was dissolved in ethanol (22 mg/ml). On the day of experiment, the dye incubation was prepared by mixing 60 µl of dye stock with 500 µl of fetal bovine serum (FBS), 500 µl of ACSF, and 310 µl of 10% cremophore-EL solution. The concentration of dye in the final solution was 0.1 mg/ml. After incubation, slices were washed for 8–10 min with ACSF and transferred to recording chamber. The temperature of the bath was maintained at 32°C during recordings and continuously supplied with carbogen-bubbled ACSF having a composition of 110 mM NaCl, 2.5 mM KCl, 1.2 mM NaH_2_PO_4_, 25 mM NaHCO_3_, 2.0 mM CaCl_2_, 2.0 mM MgCl_2_, and 20 mM dextrose. The pH and osmolarity of the solutions were adjusted to 7.3–7.4 and 297–305 mOsm, respectively. CRF ACSF was perfused into the recordings chamber for 10 min before a new set of responses were done.

Each VSDI recording was 20 s in length and consisted of two époques. The first was a 2-s recording of background activity before the stimulus followed by the stimulus application for 1 s with frequencies differing from 5 to 100 Hz. The acquisition rate was between 5 ms/frame. For each recording minimum camera saturation was set around 50% while the maximum was ∼80%. Optical recording was conducted using a CMOS camera (Micam Ultima, BrainVision, Inc.) mounted on top of an upright microscope (Fixed Stage Upright Microscope BX51WI, Olympus). The light from a 100-W halogen lamp source (HLX 64625, Microlites Scientific, Corp.) passed through an excitation filter (λ = 530 ± 10 nm). The fluorescent signals were collected and projected onto the CMOS sensor through a long pass emission filter (λ > 590 nm). A long-distance objective was used in these experiments (XLFluor 4X N.A. 0.28, Olympus). The movies were recorded and analyzed using Brain Vision Analyzer software. The acquisition settings were: 100 × 100 pixels frame size, after magnification each represented 25 × 25 µm space on the brain slice. The dye signal intensity decreases as the membrane depolarizes. However, to better match conventional recordings the signals all have been converted so that the excitatory and inhibitory signals were shown as positive and negative values. The fractional change in fluorescence signal relative to background signal (ΔF/F) was calculated for each frame of each recording. For all the recordings, we binned 5 × 5 pixels into one representative signal this bin was placed in the three areas of interest. The software locks this area into place form one recording to the next so exactly the same 5 × 5 bin is compared in each recording. The peak response was used to compare changes before and after drug application. For each recording, the bin was placed in a minimum of three regions within layer II, layer III, and the endopiriform nucleus; these were averaged to arrive at a single value that was averaged between differing slice recordings. As there was considerable variability in the magnitude of the responses from slice to slice due to differences in loading of the dye, we normalized the recordings by dividing all signals by the response to the 20-Hz stimuli. This permitted us to average the normalized responses between recordings.

A platinum/iridium electrode (Microprobes, Inc.) with a tip diameter of 200–300 µm was used to stimulate lateral olfactory tract (LOT) of PC. The stimulation of each slice was in the range of 160–200 µA, each square pulse was 2.0 ms in length. The electrode was connected to a stimulator (S88X dual output square pulse stimulator, Grass Technologies, Astro-Med, Inc.), which controlled the pulse frequency and train duration. Recordings were taken from the anteroposterior portion of the PCtx. Care was taken that all the layers of PCtx including dorsal endopiriform nucleus (DEn) and LOT (where the electrode was placed) appeared explicitly under the camera view. Drugs were applied for at least 10 min before taking a respective recording.

### *In vivo* experiments

#### Tail pinch as a model of stress

Animals were allocated to two groups as sham and TBI animals. Both groups underwent the tail pinch protocol. During this protocol, an animal was transferred to a recording chamber, and the head cap that connects the animal to recording system was placed. Following 5 min of acclimatization to the chamber environment, baseline measurement of the brain activity was taken via the implanted electrodes for 2 min (after amplification using a Grass instruments Model 55 preamplifier a Molecular Devices Digidata 1550 A/D converter sampling rate of 4 kHz). Following this, a clip of moderate strength (stress) was placed on the middle of the tails of animals. Tails were covered with a paper towel or a clean cotton rag before the paper clip was applied to prevent pain and local damage of the tissue. This stressor was applied for 2 min, and at the end of this time, the clip was removed. The recording continued for further 2 min in the absence of stress. This procedure is not considered to be painful.

#### Spectral analysis of amygdala recordings

Recordings were analyzed using MATLAB 2018a (MathWorks). To visualize the spectral responses during the stressor, the local field potential (LFP) signal was convolved with complex Morlet wavelets and then squared, thereby extracting the frequency-specific power and transforming the signal into a time-frequency representation. The Morlet wavelets were constructed with seven wavelet oscillations and center frequencies in 10-Hz steps from 1 to 500 Hz (Tallon-Baudry et al., 1997). In the spectrograms, low power is represented by blue colors and high power by dark red. The recordings were also visually checked for spikes that when filtered may produce artifacts resembling fast ripples (Bénar et al., 2010).

To examine the average responses of the two groups across the entire HFO range, the recordings were high pass filtered at >120 Hz. Power spectra were then generated for the baseline and stress response. The two spectra were then subtracted and normalized. For the five rats analyzed in each group, sham and TBI, these subtracted and normalized spectra were then averaged to generate an aggregate response for these two groups.

#### Phase amplitude coupling data analysis

Analyses were done using MATLAB 2018a (MathWorks). Raw signals were convoluted with complex Morlet wavelets with seven wave oscillations. The phase of low frequencies (wavelets centered at 1–21 Hz in steps of 2 Hz) was extracted by calculating the convolved vector in relation to the real axis. The amplitude of higher frequencies (centered at 20–500 Hz in steps of 10 Hz) was found by squaring the result of convolution (Tallon-Baudry et al., 1997). Peaks of high power (>2 SD) during the stress epoch in band-specific peaks within the wavelet spectral bandwidth were found. One-second windows centered around each peak were analyzed for their strength of phase-amplitude coupling using the modulation index (MI; Tort et al., 2010). A window length of 1 s was chosen because HFO events were ∼1 s in duration and occurred sporadically during stress. The normalized mean high-frequency amplitude value at each low-frequency phase bin, known as the *p*_j_ value, for each high-power window for all rats in each drug condition were pooled. Pooling these 1-s windows found over the 2-min stress epoch gives a more robust estimation of coupling than simply calculating the MI over a longer time window, especially during the drug condition, in which the signal-to-noise ratio is much lower due to reduction in occurrence and amplitude of HFO events. The pooled normalized values were used to calculate the MI at each frequency pairing during each drug condition and are represented in comodulograms showing preferred frequency of coupling in Figure 7. Surrogate analyses were performed via shuffling the phase of one window with the amplitude of another window (*n* = 10,000) for each frequency pairing and then calculating the MI. Also, oscillation-triggered comodulograms were created by summing 1 s of raw recordings centered around these peaks to reveal underlying rhythmic low-frequency activity (Dvorak and Fenton, 2014; Samiee and Baillet, 2017) as shown in Figure 8. The custom MATLAB scripts used are available on request.

### Reagents

Bisindolylmaleimide (BIS; PKC antagonist), PMA (PKC activator), forskolin (adenyly cyclase activator), and H89 [protein kinase A (PKA) antagonist] were obtained from Sigma-Aldrich Co. CRF and CP 154526 were obtained from Tocris Biosciences. Di-4-ANEPPS stock solution was prepared in alcohol and cremophore-EL solution, which can be stored at 4°C for two months. FBS and ACSF were added on the day of the experiment. CRF stock was dissolved in HBSS and Milli-Q water mixture. Forskolin, H89, BIS, antalarmin, and PMA stock solutions were prepared in dimethyl sulfoxide (DMSO). All the stock solutions were made 1000 times more concentrated than working concentrations.

### Statistical analysis

All statistical evaluations were done using StatView software. For the VSDI data, two-way ANOVAs were conducted to compare the neuronal responses after the application of BIS, PMA, forskolin, and H89 in the PCtx layers in response to LOT stimulation. CRF responses were analyzed by a repeated measures ANOVA in which the responses to CRF in the absence of drugs served as the within group measure. Follow-up comparisons were conducted using Fisher’s protected least significance difference test to maintain *p* at 0.05. A χ^2^ analysis was used to evaluate the seizure behaviors between sham and TBI rats. A Kolmogorov test for differences in distributions of the baseline subtracted power spectra was used to statistically analyze the sham and TBI averaged spectra (Fig. 5*D*).

## Results

**Figure 1.**
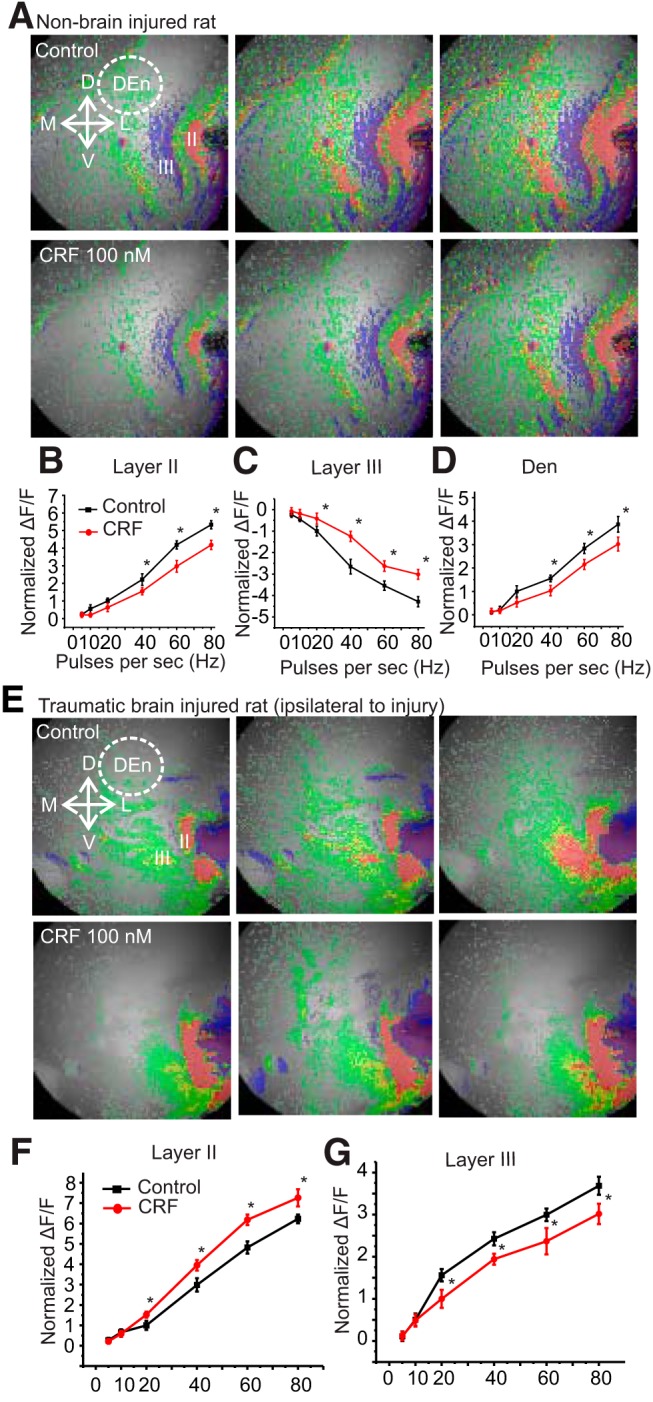
CRF application increased the activity of the PCtx in TBI rat. ***A***, Representative images showing activation of PCtx from sham rats, without (top) or with CRF (100 nM; bottom). Each image was taken at the same time interval [2 (during stimulation), 3, and 5 s]. ***B–D***, Quantification of CRFR_1_ activation shows that the activity of Layer II pyramidal cells and interneurons of DEn are decreased while the activity of Layer III interneurons is increased in non-brain-injured animals over the range of stimulation frequencies used to activate the circuit; *p < 0.05; *n* = 5 slices from four rats. ***E***, Representative images showing activation of the layers of ipsilateral PCtx from brain-injured rats, without (top) or with CRF bottom). ***F***, ***G***, Quantification of CRFR_1_ activation shows that the activity of Layer II pyramidal cells is increased while the activity of Layer III interneurons is decreased over the range of stimulation frequencies used in piriform cortical slices from TBI rats to activate the circuit; *p < 0.05. *n* = 9 slices from seven rats. No response is observed from interneurons of DEn. Arrows in panels indicate the orientation of slice. D, dorsal; V, ventral; M, medial; L, lateral. Red indicates highest ΔF/F; orange, yellow and green indicate medium ΔF/F; blue a reduction in ΔF/F.

**Figure 2.**
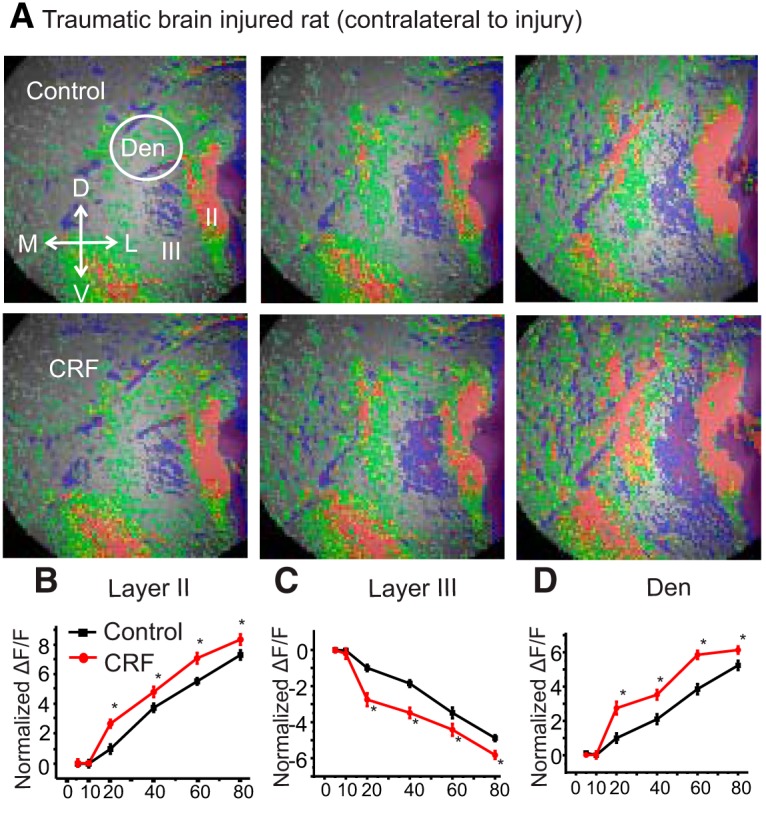
Application of CRF increased contralateral PCtx activity in brain-injured rats. CRFR_1_ mediated these effects. ***A***, Representative images showing activation of layers of contralateral piriform cortical slices from brain-injured rats, without (top) or with CRF perfusion before recording (bottom). ***B*–*D***, Quantification of CRFR_1_ activation shows that the activity of Layer II pyramidal cells and interneurons of DEn are increased while the activity of Layer III interneurons is decreased over the range of stimulation frequencies used in contralateral piriform cortical slices from brain-injured rats to activate the circuit; *p < 0.01; *n* = 8 slices from six rats.

**Figure 3.**
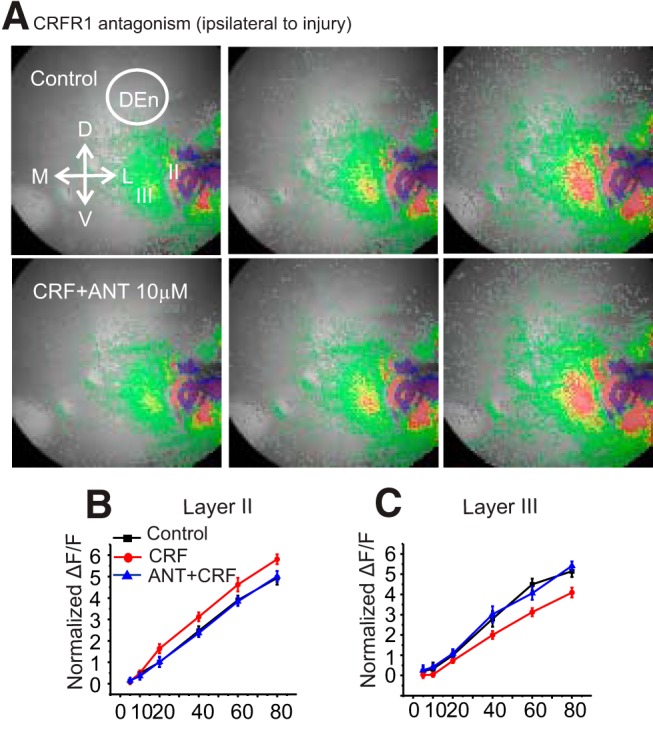
CRFR_1_ mediate CRF responses in brain-injured PCtx. ***A***, Representative images of the activation of the PCtx layers in slices at differing time points before (top) and after the application of CRF (bottom) in the presence of the CRFR_1_ antagonist antalarmin. ***B***, ***C***, Quantification of CRFR_1_ activation in the presence of antalarmin in Layer II and Layer III of ipsilateral PCtx; *n* = 6 slices from five rats.

The inhibitory effect of the stressor hormone/neurotransmitter CRF in the PCtx of rats becomes excitatory after kindling (Narla et al., 2016). We hypothesized that CRF may change its signaling after the induction of TBI and that this change may exacerbate stress responses and elicit epileptiform activity. First, we used VSDI which shows how PCtx circuitry responds to a stimulus given to the LOT and whether CRF alters this circuit activation. The PCtx is a convenient and relevant structure to assay this activity as it readily supports seizures and has strong interconnections with other limbic regions such as the amygdala, hippocampus, and entorhinal cortex (EC). Each of these are known to be involved in the stressor responses. In a previous report, it has been shown (Birjandian et al., 2013) that in the non-brain-injured rat, the network response is characterized by three phases of the response in Layers II, III, and DEn (Fig. 1*A*, top row). Layer II is activated first followed by the activation of the DEn and then the deactivation of the Layer III. This is done by stimulation of LOT with different frequencies ranging from 5 to 80 Hz (Fig. 1*B–D*). In sham rats, CRF reduced the excitability of Layer II and DEn and the deactivation of Layer III (Fig. 1*A*, bottom row, *B–D*). However, after TBI, CRF alters this network activation and increases the excitability but with important differences in the pattern of activity seen in non-injured animals. Ipsilateral to the injury there was little to no DEn response observed, and the deactivation of Layer III neurons flips polarity becoming activated (Fig. 1*E*, top row, *F–H*). We have provided evidence in the past that this deactivation is caused by the disinhibition of the Layer III interneurons and relies on the activation of neurons in the DEn. Despite the activation of the interneurons in Layer III, CRF application still increased the excitability of Layer II pyramidal cells which are the primary projection neurons of this circuit (Fig. 1*E*, bottom row, *F–H*). Thus, after brain injury, CRF increased circuit activation, an effect opposite to non-brain-injured rats. CRF was also able to increase circuit activation on the contralateral PCtx. However, the pattern of circuit activation was not the same as on the contralateral side and was similar to sham controls where Layers II, III, and the DEn were all involved (Fig. 2). Nevertheless, CRF increased the excitability in these assays as well. The activity of CRF was blocked by the CRFR_1_ antagonist, antalarmin (100 nM; Fig. 3), on the both ipsilateral and contralateral sides. Previous work has shown that the CRF antagonists have no effect on their own in this assay (Narla et al., 2016).

**Figure 4.**
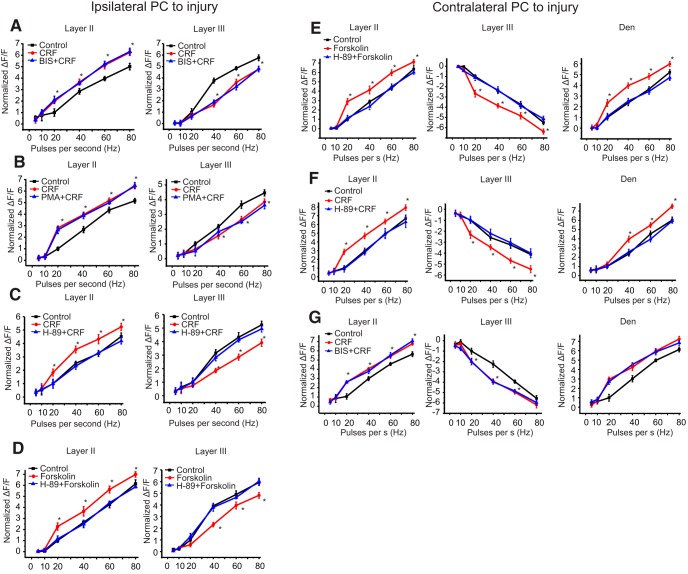
PKA and PKC antagonism in ipsilateral and contralateral PCtx in the presence of CRF. ***A***, Quantification of the effect of BIS on CRFR_1_ activation in ipsilateral piriform cortical slices from brain-injured animals; *p < 0.05, *n* = 8 slices from eight rats/group. ***B***, Quantification of the effect of the PKC inhibitor, PMA, on CRF-mediated activation of CRFR_1_ in ipsilateral piriform cortical slices from brain-injured animals; *p < 0.05, *n* = 9 slices from eight rats/group. ***C***, Quantification of the effect of the PKA antagonist H89 on CRF-mediated activation of CRFR_1_ in ipsilateral piriform cortical slices from brain-injured animals; *p < 0.05, *n* = 9 slices from seven rats/group. ***D***, Quantification of the effect of forskolin, an adenylate cyclase activator with or without subsequent administration of H89 on ipsilateral piriform cortical slices from brain-injured animals; *p < 0.05, *n* = 8 slices from six rats/group. ***E***, Quantification of the effect of forskolin (an adenylyl cyclase activator) with or without subsequent administration of H89, on CRFR_1_ activation in contralateral piriform cortical slices from brain-injured animals; *p < 0.05, *n* = 7 slices from six rats/group. ***F***, Quantification of the effect of the PKA inhibitor H89 on CRF-mediated activation of CRFR_1_ in contralateral piriform cortical slices from brain-injured animals; *p < 0.05, *n* = 6 slices from six rats/group. ***G***, Quantification of the effect of the PKC antagonist BIS on CRF-mediated activation of CRFR_1_ in contralateral piriform cortical slices from brain-injured animals; *p < 0.05, *n* = 6 slices from six rats/group.

In the TBI rat, inhibition of PKC by BIS or its activation by PMA did not block or mimic the effects of CRF (Fig. 4*A–D*). However, forskolin, an activator of adenylyl cyclase that mimics the effect of activated Gα_s_, produced responses similar to that of CRF. Forskolin activity was antagonized by the PKA inhibitor H89 (Fig. 4*E*,*F*). This is in contrast to sham control rats where CRFR_1_ couples to Gα_q/11_ and thus activates PKC (also see Narla et al., 2015, 2016). Thus, TBI alters the effects of CRF on network behavior likely by an alteration in G-protein coupling.

**Figure 5.**
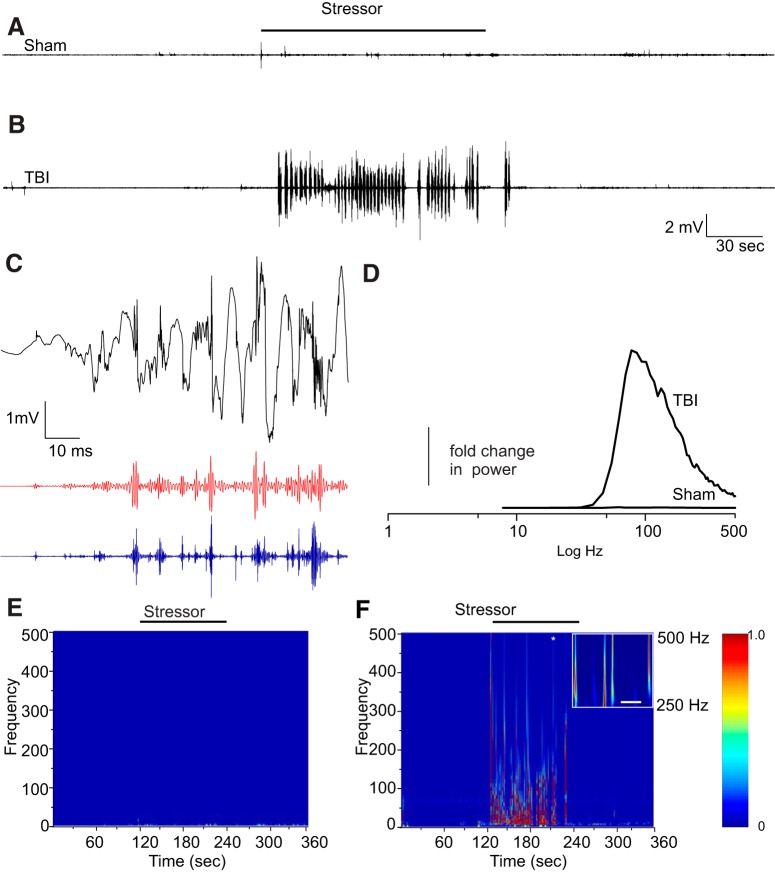
Stress induces a larger electrographic response in the amygdala of TBI than in sham rats ***A***, Recording of TBI rat amygdala response from a sham operated rat showed only small electrographic response to a tail inch stress, while in ***B***, TBI-injured rats showed a large increase in electrical activity. ***C***, A 100-ms sample of a response where ripples (red band pass filtered >120 < 250 Hz) and fast ripples (blue high pass filtered >250 Hz) are clearly visible. ***D***, Mean normalized power spectra of sham (*n* = 5) and TBI (*n* = 5). HFOs compared to lower frequencies showed a 2.5-fold change in power in TBI rats, but little change in sham rats. ***E***, ***F***, A comparison of frequency content of sham rats' response using Morlet wavelet analysis shows that sham rats have only small responses to the stressor, while in TBI rats, large increases in electrical activity during stressor. The inset Morlet using a smaller window shows well-resolved fast ripple activity (* marks approximate time from which the window was obtained; white scale bar is 100 ms).

In contrast to mice, rats in this TBI model rarely develop spontaneous seizures (Bolkvadze and Pitkänen, 2012). Nevertheless, as reported in the past (Pitkänen et al., 2009, 2014), injured rats do have epileptiform activity. We wanted to determine whether TBI rats would respond to an acute stressor differently than sham rats and whether electrophysiological responses from the stressor responsive nuclei, the amygdala (which is strongly connected to the PCtx), may be affected. We used the acute tail pinch to deliver a stressor to rats while recording from the amygdala (Gibb et al., 2008). A trial began with a baseline recording for 2 min in the absence of the stressor followed by 2-min recording in the presence of the stressor ending by a 2-min recording in the absence of the stressor. We were also particularly interested to see whether HFOs (ripples > 120 < 250 Hz) and fast ripples (>250 Hz) became more prevalent as the increased occurrence of latter wave forms are associated with epileptic activity (Jefferys et al., 2012). We found that in sham rats the responses to this stressor were mild (Fig. 5*A*) and high-frequency content was much lower (Fig. 5*E*), although the rats were equally agitated and attempted to remove the clip from their tail in response to the stressor. In Figure 5*B*, we show a typical electrical response from a TBI rat recorded from the amygdala during a tail pinch stressor. This electrophysiological response in the TBI rats was accompanied by stage 2–3 seizures (Racine scale; av 2.5 ± 0.4 SD, *n* = 5 rats; Racine et al., 1988). Sham rats did not have any seizure behaviors (*p* < 0.01, χ^2^ analysis). In Figure 5*C*, we show a sub sample of the response in Figure 5*B* (150 ms). The unfiltered sample is black, while the band passed 120–250 Hz is red, and high pass filtered >250 Hz is blue, showing both ripples and fast ripples, respectively. In Figure 5*D*, we show the averaged power spectra of the high passed filtered recordings (>120 Hz) from five sham TBI and five TBI rats. This was done by subtracting the power spectra of the baseline from the spectra during stressor responses. The baseline corrected spectra were then normalized and averaged. We found little or no increase in power at high frequencies in the sham rats, but in TBI rats, the power increased by ∼2.5-fold (particularly above 250 Hz, the distributions of these two spectra showed they were not the same *p* < 0.001, Kolmogorov–Smirnov test**).** A spectral analysis of the entire response using a Morlet wavelet transform revealed a small increase in power at frequencies up to 100 Hz in the sham rats but HFOs (ripples and fast ripples) were rare, although some were observed (Fig. 5*E*). This was very different in TBI rats (Fig. 5*F*), where both kinds of ripples occurred during the application of the stressor. Figure 5*F*, inset, shows that the wavelet analysis over a 500-ms window.

**Figure 6.**
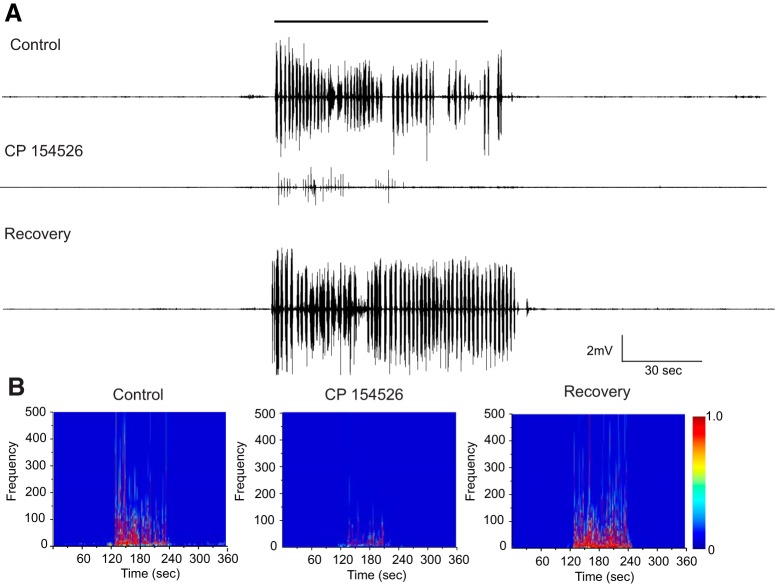
CRFR_1_ antagonism *in vivo* in brain-injured animals reduced electrographic response and HFOs during stress. ***A***, Tail pinch response from a TBI rat followed by the response after the application of CP 154526, a CRFR_1_ antagonist, which is followed by a recovery trace after 24 h of drug washout. Duration of stress is indicated by the bar placed above the control response which was for 2 min. ***B***, Morlet spectrograms which correspond to the traces shown in ***A***.

In Figure 6, we show a typical electrophysiological response from a TBI rat and its attenuation by the CRFR_1_ antagonist CP 154526 (1.5 h after an intraperitoneal injection of 50 mg/kg). All rats also exhibited no seizure behavior. The accompanying Morlet wavelet analyses showed a marked attenuation of activity in both the high and low frequency 3 h after the injection and this was reversed 24 h later.

**Figure 7.**
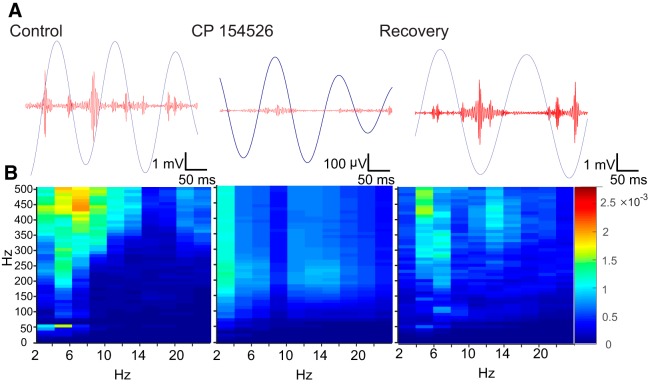
CRF mediates the phase/amplitude coupling during stressor. ***A***, upper traces, Examples of fast ripple activity and θ band in control, CP 154526, and after 24-h drug wash out from TBI rats under stress filtered into θ (3–8 Hz, blue) and fast ripples (250–500 Hz, red) bands. ***B***, Comparison of MIs between low-frequency phase on *x*-axis and higher frequency amplitude between 250 and 500 Hz on *y*-axis. Fast ripples (>250 Hz) are significantly and highly coupled over the frequency bins corresponding to θ rhythm. Ripple activity is also coupled to a lesser extent in the same range. This relationship is abolished by CRFR_1_ antagonism. Very low-frequency activity (2–4 Hz) is apparently coupled over a wide range of high-frequency activity. The MI values corresponding to this coupling during antagonist are below chance. After 24 h, the PAC returned.

We next examined whether the high-frequency activity was phase/amplitude locked to any particular frequencies. In Figure 7, we show a summary of the phase amplitude coupling (Tort et al., 2010) between low-frequency activity (binned on *x*-axis) versus high frequencies (binned on the *y*-axis) in the signals before, during, and after CRFR_1_ antagonist treatment. The averaged MIs from all five rats are shown in this analysis and are color coded as a heat map on the left. The control responses showed that in range of 4–8 Hz, there was high coupling in both the ripple and fast ripple bands. This was particularly prominent between for the fast ripple whose MIs were the largest (MI = 2.05 × 10^−3^ vs surrogate MI = 5.47 × 10^−5^). In the CP 154526-treated rats, no coupling was only observed between the θ band and high-frequency bands. These MIs calculated in the presence of antagonist were below chance, as calculated by surrogate analyses of shuffling (MI = 1.28 × 10^−3^ vs surrogate MI = 4.11 × 10^−3^). After recovery, the coupling between 4 and 8 Hz and the ripple and fast ripple bands were again present (MI = 1.56 × 10^−3^ vs surrogate MI = 2.77 × 10^−5^). There was no preferred phase angle in both control and washout recordings.

**Figure 8.**
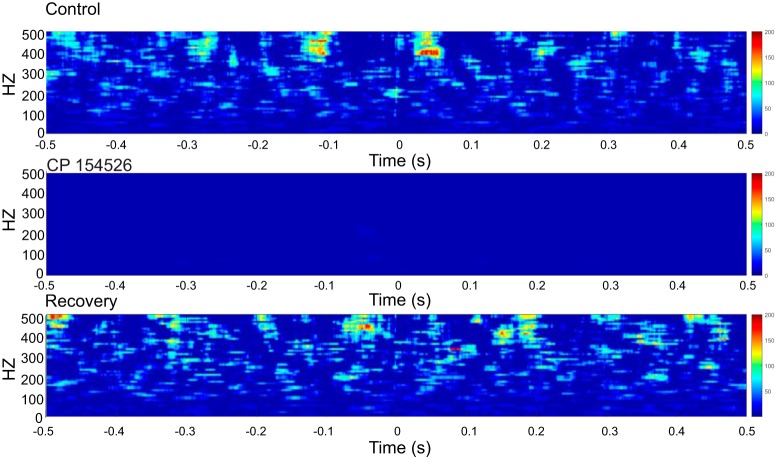
Phase-amplitude coupling from data recorded during a stressor in TBI rats. The oscillation-triggered comodulogram shows coupling between fast ripples and an underlying 5-Hz oscillation without antagonist and after washout of antagonist. During antagonist, destructive interference of the pooled LFPs shows no significant coupling.

Finally, to confirm this analysis, we also calculated an oscillation-triggered comodulogram (Dvorak and Fenton, 2014; Samiee and Baillet, 2017) for the pooled data before, during, and after CRFR_1_ antagonism. The crests of fast ripple activity aligned at a slow oscillation of ∼0.2 s between crests, or a 5-Hz wave (θ). This analysis confirms the observations using the MI described by Tort et al. (2010).

## Discussion

TBI is a major risk factor in the development of epilepsy (Xu et al., 2017). PTSD and TBI are often comorbid as brain injuries are often associated with traumatic experiences (Ohry et al., 1996; Hickling et al., 1998; Levin et al., 2001; Greenspan et al., 2006; Gaylord et al., 2008; Hoge et al., 2008; Bryant, 2011). To date, a few mechanisms have been proposed to account for the high association of PTSD and the development of epilepsy such as upregulated glutamate neurotransmission, axonal damage, and compromised interneuronal circuits (Guerriero et al., 2015). CRF has been implicated in several brain disorders such as anxiety, stress, depression, and neuropathic pain. Clinical studies have shown that increased concentrations of CRF in the CSF are associated with PTSD (Post et al., 1982) and several studies have found altered hypothalamic pituitary adrenal (HPA) axis and CRF function in PTSD (Mason et al., 1986; Smith et al., 1989; Pitman and Orr, 1990; Yehuda et al., 1993). Consistent with these reports, studies in humans showed a chronic increase in CRF release following stress exposure (Dallman and Jones, 1973). In TBI rats, we showed that CRF exhibited excitatory effects in the PCtx, a region that has strong interconnections with most limbic regions. Excitatory activity of CRF was wide spread, occurring on both the injured and uninjured sides. Therefore, these changes in CRF activity have a wide impact that either originate in the PCtx or involve changes that associate other limbic regions to produce the electrophysiological responses we observed in the amygdala.

Importantly, our observations indicate that pathologic mechanisms that accompany brain injury make the brain susceptible to stressor-induced increases in the excitability of the amygdala. In particular, the phase coupling between the pathophysiological HFOs and θ oscillations seem important as this coupling may aid in the spread of the high-frequency activity contributing to epileptogenesis. Indeed, it has been noted in previous studies that HFOs have a surprising ability to spread and exhibit high coherence across much larger volumes than one might expect (Haufler and Pare, 2014). These findings also provide insight into why those who have suffered TBI and PTSD have a 3-fold increase in the incidence to develop epilepsy, which is often pharmacoresistant (Hitiris et al., 2007).

For over 15 years, HFOs have been the subject of great interest. Here, we have examined HFOs that occur above 120 Hz (above high γ) to the 500 Hz. HFOs (ripples) between 120 and 250 Hz are thought to be, in general, physiologic, whereas those occurring above 250 Hz (fast ripples) are generally considered to be pathologic HFOs (pHFOs), supporting the generation of epileptic seizures (Bragin et al., 1999; Jefferys et al., 2012). This view is by no means settled and other phenomena may also need to be considered as contributing to the pathophysiological endpoints. These include whether they are bursting (like observed here) or steady state HFOs, phase locked to a behavioral or electrical event and their location (Jefferys et al., 2012). Also, it has been reported that ripples are associated with epileptic behavior in animal models (Shiri et al., 2016) as well as in humans (Weiss et al., 2016). The other issue is whether pHFOs are an epiphenomena or causative of the epileptic state. Evidence that the latter may be true comes from work showing that during the transition period from the latent to the chronic phase in the pilocarpine model of mesial temporal lobe epilepsy, there is an increased incidence of HFO activity in the EC and CA3 region of the hippocampus (Salami et al., 2014) and the block of their occurrence with the AED levetiracetam impeded seizure genesis. Here, we have recorded HFOs from the amygdala, a region of the brain that readily supports the development of seizures (McIntyre et al., 2002). Thus, the occurrence of fast ripples in this region would be predicted to be supportive of the development of seizure activity in PCtx. Previously, HFOs have been isolated in the amygdala and associated limbic regions (DEn, central amygdala, EC, and PCtx; Ponomarenko et al., 2003; Haufler and Pare, 2014; Salami et al., 2014). In general, these studies have found that HFOs occur regionally in a highly synchronous manner so that HFOs occurring in one region are highly correlated with occurrences in other regions. In fact, it was noted that an apparently unusual property of HFOs relative to all other fast frequency (γ) LFPs was their high coherence (Haufler and Pare, 2014), although this was less when ipsilateral and contralateral structures were compared. LFP coherence usually decreases with distance, and this reduction is quite steep for their fast components (Steriade et al., 1996; Collins et al., 2001). In addition, the main difference between what was observed in past reports and our findings is that the fast ripples recorded here were highly phasic locked to θ oscillations. A recent study has shown that high-power low-frequency oscillations coupled to HFOs could be readily identified in a brain region of patients with pharmacoresistant epilepsy and when this region was subsequently removed, better clinical outcomes were seen (Cotic et al., 2016). This supports the idea that the HFO/θ cross-frequency coupling seems to be highly epileptogenic.

While it clear that stress generates HFOs, how this occurs is not known. It seems likely that CRF is influencing the activity of the local circuit neurons to produce these network effects but exactly how this occurs is not known. CRF has a wide range of activity on both excitatory and inhibitory neurons of the PCtx in naïve rats. In particular, it has highly complex effects on interneurons. In some cases, CRF application converted slow firing interneurons to fast firing ones, in others fast firing was converted to slow while on another population CRF had no effects (Narla et al., 2015). Further studies will be required to provide insight into how CRF affects these neurons after TBI.

There is also an added complexity here, as ipsilateral to the injury, interneurons of Layer III became excitable while the interneurons of DEn were silent. We have ruled out cell loss and the degeneration of axons as a cause of this change in the activation pattern (unpublished observations). One possibility that may explain this is the observation that suggests interneurons become functionally silent after an insult. Evidence for this interpretation comes from a study where the perforant path model of temporal lobe epilepsy was used. Sloviter (1987) described disruption of functional inhibition in the presence of viable interneurons and functionally active GABA_A_ receptors in the dentate gyrus and CA1 areas of hippocampus (Sloviter, 1987, 1991). Similar observations were also observed by two other studies showing no loss of GABAergic interneurons (Bekenstein and Lothman, 1993; Lothman et al., 1996) but a reduction in inhibitory activity. This paradox has been described as a “dormancy of interneurons” where the inhibitory cells became quiescent and disconnected. Similar observations were reported in tetanus toxin model of epilepsy where evidence for the existence of functionally dormant interneurons was shown (Jefferys and Traub, 1998). To what degree this may occur in the BLA is not known.

Our current findings suggest that CRFR_1_ antagonists may have a clinical importance as antiepileptic drugs. CRFR_1_ antagonists have long been under investigation for treating stress related disorders such as anxiety and depression, although the results have not been promising (Zorrilla and Koob, 2004; Valdez, 2009). No clinical study has investigated or argued for the use of CRFR_1_ antagonists in post TBI to prevent epilepsy. Also, it has been observed that exogenously applied CRF induced excitotoxicity of interneurons (Aldenhoff et al., 1983; Bishop and King, 1992), which suggests that CRFR antagonists may be effective in providing neuroprotection in the hippocampus following seizure (Maecker et al., 1997) and after cerebral ischemia (Lyons et al., 1991).

In summary, our findings provide a mechanism by which individuals affected by TBI develop stress-associated epilepsy. Molecular mechanisms following brain injury alter the function of stress hormone/neurotransmitter that causes a widespread increase in excitability. In particular, the generation of fast ripples during the response and their phase coupling to the θ oscillations could be particularly epileptogenic.

## Synthesis

Reviewing Editor: Quentin Pittman, University of Calgary Decisions are customarily a result of the Reviewing Editor and the peer reviewers coming together and discussing their recommendations until a consensus is reached. When revisions are invited, a fact-based synthesis statement explaining their decision and outlining what is needed to prepare a revision will be listed below.

The authors have substantially improved the manuscript
